# Lack of Cross-Modal Effects in Dual-Modality Implicit Statistical Learning

**DOI:** 10.3389/fpsyg.2018.00146

**Published:** 2018-02-27

**Authors:** Xiujun Li, Xudong Zhao, Wendian Shi, Yang Lu, Christopher M. Conway

**Affiliations:** ^1^School of Psychology and Cognitive Science, East China Normal University, Shanghai, China; ^2^Department of Psychology, School of Education, Shanghai Normal University, Shanghai, China; ^3^NeuroLearn Lab, Department of Psychology, Georgia State University, Atlanta, GA, United States; ^4^Neuroscience Institute, Georgia State University, Atlanta, GA, United States

**Keywords:** implicit statistical learning, cross-modal learning, modality-specific, multimodal input, dual-modality

## Abstract

A current controversy in the area of implicit statistical learning (ISL) is whether this process consists of a single, central mechanism or multiple modality-specific ones. To provide insight into this question, the current study involved three ISL experiments to explore whether multimodal input sources are processed separately in each modality or are integrated together across modalities. In Experiment 1, visual and auditory ISL were measured under unimodal conditions, with the results providing a baseline level of learning for subsequent experiments. Visual and auditory sequences were presented separately, and the underlying grammar used for both modalities was the same. In Experiment 2, visual and auditory sequences were presented simultaneously with each modality using the same artificial grammar to investigate whether redundant multisensory information would result in a facilitative effect (i.e., increased learning) compared to the baseline. In Experiment 3, visual and auditory sequences were again presented simultaneously but this time with each modality employing different artificial grammars to investigate whether an interference effect (i.e., decreased learning) would be observed compared to the baseline. Results showed that there was neither a facilitative learning effect in Experiment 2 nor an interference effect in Experiment 3. These findings suggest that participants were able to track simultaneously and independently two sets of sequential regularities under dual-modality conditions. These findings are consistent with the theories that posit the existence of multiple, modality-specific ISL mechanisms rather than a single central one.

## Introduction

Human learners show sensitivity to environmental regularities across multiple perceptual modalities and domains even without being aware of what is learned (Aslin and Newport, 2009; Emberson and Rubinstein, 2016). This ability, referred to as implicit statistical learning (ISL), is a ubiquitous foundational cognitive ability thought to support diverse complex functions (Guo et al., 2011; Thiessen and Erickson, 2015).

A current debate in this area of research concerns the mental representations resulting from ISL. The nature of these mental representations is important for revealing the characteristics of the mechanisms underlying ISL (Cleeremans and Jiménez, 2002; Fu and Fu, 2006; Li and Shi, 2016). In the classic study of implicit learning, Reber (1967) demonstrated ISL in participants who were exposed to letter strings generated from an artificial grammar. In these experiments, letter strings obeyed the overall rule structure of the grammar, being constrained in terms of which letters could follow which other letters. Participants not only showed evidence of learning this structure implicitly, but also could apparently transfer their knowledge of the legal regularities from one letter vocabulary (e.g., M, R, T, V, X) to another (e.g., N, P, S, W, Z) as long as the underlying grammar used for both was the same. This effect has been replicated many times, with transfer being demonstrated not just across letter sets (Shanks et al., 1997), but also across perceptual modalities (Tunney and Altmann, 2001). The transfer effects in artificial grammar learning (AGL) are usually explained by proposing that the learning is based on abstract knowledge, that is, knowledge that is not directly tied to the surface features or sensory input (Reber, 1989; Altmann et al., 1995; Shanks et al., 1997; Peña et al., 2002). An additional characteristic of ISL is that it occurs with perceptually diverse input, including linguistic stimuli, tone stimuli, visual scenes, geometric shapes, color stimuli, and motor responses (Saffran et al., 1999; Fiser and Aslin, 2002; Kemény and Lukács, 2011; Durrant et al., 2013; Goujon and Fagot, 2013; Guo et al., 2013). Importantly, the same ISL phenomenon appears to be observed regardless of the nature of the input patterns. Given that ISL occurs with perceptually diverse input, it is possible that what underlies ISL is a single, central mechanism that treats all types of input stimuli (e.g., tones, shapes, and syllables) as equivalent beyond the statistical structure of the input itself.

However, there is evidence contrary to this view, suggesting that ISL is not neutral to the input modality but rather is rooted in modality-specific, sensorimotor systems. First, demonstrations of transfer of knowledge does not necessarily mean that the acquired knowledge is amodal. What is learned may be the surface characteristics of the stimuli but a separate, higher-level process may form mappings between the different types of input, allowing above-chance performance with the new input (Redington and Chater, 1996). Consistent with this view, a recent study showed that transferring knowledge to a new stimulus set in an AGL paradigm required working memory resources; when memory resources were depleted using a dual-task manipulation, no transfer effects were observed despite learning of the regularities occurring (Hendricks et al., 2013). Second, although ISL can occur with different types of stimuli, this does not necessarily indicate that ISL is subserved by a single, domain-general mechanism that applies across a wide range of tasks, inputs, and domains. Instead, it is just as possible that there may exist multiple parallel subsystems, each relying on similar computational algorithms, which can process and learn the underlying structure in various stimuli, (e.g., Chang and Knowlton, 2004; Conway and Christiansen, 2005; Conway and Pisoni, 2008; Goujon and Fagot, 2013; Frost et al., 2015). For example, Chang and Knowlton (2004) found that ISL was sensitive to stimuli features, and the changes in fonts could affect the ISL performance of the letter strings. This finding appears to indicate that at least some of the learned knowledge is modality or stimulus specific. In addition, using vibration pulses, pictures, and pure tones as experimental materials, Conway and Christiansen (2005) compared tactile, visual, and auditory ISL and found modality constraints affecting ISL across the senses, with auditory ISL showing better performance than both tactile and visual learning (see also Conway and Christiansen, 2006, 2009). Similarly, Emberson et al. (2011) presented visual and auditory input streams under different timing conditions (fast or slow presentation rates). The results showed that auditory ISL was superior to visual learning at fast rates, but the opposite was true at slower presentation rates, suggesting the existence of modality constraints affecting learning.

The studies reviewed to this point relied on comparisons across individual modalities. However, the perceptual environment is rarely limited to one modality or a single information stream (Stein and Stanford, 2008), and learners often face multiple potential regularities across modalities at the same time. Research in the area of multisensory integration suggests that to some extent, information can be processed separately and in parallel across different perceptual modalities. For instance, participants can monitor simultaneously visual and auditory inputs in different spatial locations without a behavioral deficit under conditions of divided attention (Santangelo et al., 2010). In addition, findings from working memory research suggests that when information is presented in both visual and auditory-verbal formats, the information is encoded separately and yet a facilitative effect is also observed in bimodal formats (e.g., audiovisual stimuli), leading to improved memory (Mastroberardino et al., 2008). Although these studies demonstrate the manner in which multimodal input streams are processed by attentional, perceptual, and memory mechanisms, it is currently unclear to what extent ISL can support such processing demands.

It is important therefore is to explore the degree to which multimodal information streams are processed independently or are integrated together to support implicit learning (e.g., Sell and Kaschak, 2009; Cunillera et al., 2010; Mitchel and Weiss, 2010, 2011; Thiessen, 2010; Shi et al., 2013; Mitchel et al., 2014; Walk and Conway, 2016). The extent to which simultaneous multisensory input are processed independently rather than being integrated together provides perhaps the strongest support for the existence of multiple, modality-specific mechanisms of ISL. That is, learning of multiple input streams in parallel does not seem feasible for a single central learning mechanism; only if multiple learning mechanisms exist could parallel input streams be learned and represented independently of one another.

Conway and Christiansen (2006) assessed multistream learning in a series of three experiments. In Experiment 1, participants were exposed alternately with auditory sequences produced from one artificial grammar and visual sequences generated by a second grammar. In the test phase, new sequences were generated from each grammar; crucially, for each participant, all sequences from both grammars were instantiated only visually or auditorily. The results revealed that participants only endorsed a sequence as “grammatical” if the sensory modality matched the grammar that it was paired with during the learning phase. These findings suggest that ISL is closely bound to the input modality in which the regularities are presented, rather than operating at an abstract level. Johansson (2009) extended these findings by increasing the amount of exposure during the learning phase, believing that this would be more likely to result in formation of abstract representations. The results were still consistent with stimulus-specific, not abstract, representations.

However, due to the crossover design used (Conway and Christiansen, 2006; Johansson, 2009), these two studies were not able to examine the learning of cross-modal sequences at the same time in a strict sense. That is, the visual sequences and auditory sequences were interleaved and alternated with one another rather than being presented concurrently. Therefore, in order to provide evidence that multiple sensory modalities can be used to learn sequential regularities simultaneously and independently, a different type of design is necessary.

In an initial study using a dual-modality design, Shi et al. (2013) presented participants with visual and auditory sequences simultaneously, examining the degree to which multimodal input sources are processed independently. They found that the participants could acquire the regularities presented simultaneously regardless of the grammatical rules being the same or different, and there were no significant differences between unisensory and multisensory conditions. That is to say, audiovisual presentation of the same regularities did not show an enhanced ISL effect one might expect if there was a single learning system integrating the perceptual inputs. Similarly, audiovisual presentation of different grammars at the same time also did not show an interference effect, suggesting that the learning of two sets of regularities presented in two different perceptual modalities can occur independently and in parallel.

In the Shi et al. (2013) experiments, the audiovisual sequences consisted of nonsense syllables and color pictures paired together. The absence of enhanced or decreased ISL during the cross-modal presentation conditions may be due to the nature of the stimuli. Specifically, Conway and Christiansen (2006) found that when two input streams following different grammatical rules were presented in separate perceptual dimensions (e.g., unfamiliar shapes and nonsense syllables), participants were able to demonstrate learning of the two grammars. However, when the two input streams containing different grammatical rules were presented in the same perceptual dimension (e.g., two different sets of nonsense syllables or two different sets of unfamiliar shapes), participants were not able to learn both sets of regularities (Conway and Christiansen, 2006). Therefore, the perceptual characteristics of the stimuli appear to play an important role in cross-modal ISL.

Based on these findings, we conjectured that cross-modal effects would be more likely to occur if the two input streams contained high perceptual overlap, namely, visual and auditory stimuli that referred to the same objects. Specifically, in this study, we used animal pictures as the visual stimuli and their names as the auditory stimuli. Using pictures of animals and their names presented concurrently should provide the strongest test of parallel learning mechanisms. That is, if learning of the grammatical patterns operates independently over visual and auditory input, then concurrent presentation of animal pictures with their auditory names should show no behavioral facilitation. On the other hand, if learners are representing the visual pictures and auditory names as a single perceptual event (e.g., a multimodal percept that includes the picture of the animal tied to its name), then we might expect to observe better learning under concurrent compared to unimodal presentation. Similarly, if the animal pictures and animal names are presented in sequences generated from two different artificial grammars at the same time, this would be expected to result in an interference effect if learners are trying to integrate the pictures with the names. But if no interference is observed under such seemingly difficult learning conditions, this would be strong evidence for parallel, independent learning mechanisms.

The objective of this study therefore is twofold. First, we tested whether multistream, cross-modal ISL results in increased learning when the auditory and visual stimuli provide redundant information. Second, we explored whether multistream cross-modal ISL results in decreased learning when the auditory and visual stimuli provide conflicting information. The answers to these questions will ultimately allow us to determine whether the cross-modal patterns were processed independently within each modality or whether the perceptual modalities are integrated together during the learning process.

## Experiment 1A: Unimodal Visual ISL

Visual ISL of a single modality was tested, and used to establish a baseline level of performance for comparison with dual-modality ISL in subsequent experiments.

### Method

#### Subjects

Twenty-two Chinese graduate students with normal hearing and normal or corrected-to-normal vision were recruited from Shanghai Normal University via an advertisement (Age range = 23–29; Mean age = 25.75; Females = 13). The decision of the sample size of 22 was predetermined by priori power analysis, based on the G^∗^Power package version 3.1.9.2, which indicated the sample size > 19 adequately makes a moderate experimental effect (Cohen’s *d* = 0.8) being detectable with power = 0.9 at alpha = 0.05. None of them had ever participated in any type of cognitive experiments. This study was carried out in accordance with the recommendations of the Ethics committee of the Shanghai Psychological Society with written informed consent from all subjects. All subjects gave written informed consent in accordance with the Declaration of Helsinki, and they were paid for their participation. The protocol was approved by the Ethics committee of the Shanghai Psychological Society.

#### Materials

**FIGURE 1:**
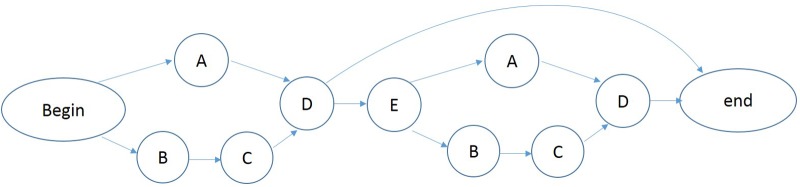
Artificial grammar 1.

An artificial grammar (see **Figure 1**) (Christiansen et al., 2012) was used to produce a set of sequences containing between five to seven elements. Each letter of the grammar was mapped onto an animal vocabulary including tiger, lion, elephant, horse and goat. The grammar determined the order of sequence elements drawn from five different categories of stimulus tokens. Two categories, A and B, each contained a single token, a tiger (A) and a lion (B), respectively. The C category consisted of two tokens, a black elephant (C_1_) and a gray elephant (C_2_). The D and E categories each contained three tokens, a white horse (D_1_), a black horse (D_2_), and a gray horse (D_3_); and a white goat (E_1_), a black goat (E_2_), and a gray goat (E_3_), respectively. There were a total of 10 tokens distributed over the ten stimuli.

A large number of sequences can be generated according to this artificial grammar. We used 48 legal sequences that were generated from the grammar for the acquisition phase (see Appendix A, Grammar 1). The test set (see Appendix B) consists of 80 novel legal and 80 illegal sequences. The illegal sequences each began with a legal element, followed by several illegal transitions and ending with a legal element. For example, the illegal sequence B–C_1_–E_2_–A–D_1_–D_1_ begins and ends with legal elements (B and D, respectively) but contains two illegal interior transitions.

**FIGURE 2:**
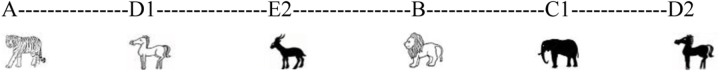
Example of sequence.

A possible token sequence resulting from this artificial grammar could be A-D_1_-E_2_-B-C_1_-D_2_.which corresponds to a tiger, white horse, black goat, lion, black elephant, and black horse (see **Figure 2**).

#### Procedure

The experiment consisted of two phases: a learning phase and a testing phase. At the beginning of the acquisition phase, participants were told a story adapted from Rosas et al. (2010) but presented in Chinese: “*Thomas travels along his country in a train carrying his circus. They arrived in a total of 144 cities. Whenever they arrive in a city, Thomas makes his animals perform. Next, you’ll see sequences of animals on the computer screen. They are the appearance order of animals performing at each city. Please remember these sequences and afterward you will take a test*.”

The participants were not told that the sequences had been produced according to an artificial grammar. The sequences were presented one at a time to each participant. Each animal picture in the sequences lasted for 1000 ms with 300 ms inter-animal intervals. A 2000 ms pause occurred between the first and second sequences. Each of the 48 learning phase sequences was presented three times in random order for a total of 144 trials.

After the familiarization phase, participants were presented with the testing phase. The testing phase started with the following story: “*The animals appearing in the orders you have observed were generated according to a set of complex rules that determined the order of the animals within each city. Thomas will travel along another country, so animals appearing in new orders have been produced. Some of these new orders conform to the same rules as before, the others are different. Only the orders conforming to the same rules would be allowed to perform on stage. Next you will see the animals appearing in new orders. Your task is to determine if the orders conform to the same rules as before by pressing one of two buttons marked YES and NO without feedback. You need to respond quickly and accurately.*” Then, the 160 test sequences were presented in random order to the participants. The presentation time of the test sequences was the same as that used during the acquisition phase.

After completing the experiment, the participants were debriefed about their explicit knowledge of the rules as well as any particular strategies that they might have used during both the training and testing phases.

### Results and Discussion

The mean test accuracy in Experiment 1A was 122 out of 160 (76%), with a standard deviation of 24. A one-sample *t-*test indicated that performance for the unimodal visual ISL task was significantly above chance, *t*(21) = 8.26, *p* < 0.001, *d* = 1.73.

## Experiment 1B: Unimodal Auditory ISL

Auditory ISL of a single modality was tested and used to establish a baseline level of performance for comparisons with dual-modality ISL in subsequent experiments.

### Method

#### Subjects

Twenty-four new Chinese graduate students were recruited in the same manner as in Experiment 1A (Age range = 23–29; Mean age = 25.88; Females = 14). A parallel power analysis with Experiment 1A was applied, and yielded a result that a sum of 46 participants was acquired to detect a large condition effect across groups (Cohen’s *d* = 1) at level of power = 0.9 and alpha = 0.05. Thus, subject number was 24, generated by subtracting subject number used in Experiment 1A (*N* = 22) from 46.

#### Materials

The experimental materials were the same as Experiment 1A except that animal names were presented in the auditory modality instead of pictures. Animal names recorded by a female, native Chinese speaker. They were presented via headphones.

#### Procedure

The procedure was identical to Experiment 1A, except that all references to animal pictures were replaced with sounds.

### Results and Discussion

The mean test accuracy in Experiment 1B was 118 out of 160 (74%), with a standard deviation of 25. A one-sample *t-*test indicated that performance for auditory ISL was significantly above chance, *t*(23) = 7.06, *p* < 0.001, *d* = 1.50. There were no statistical differences between visual and auditory levels of learning, *t*(44) = 0.59, *p* = 0.56.

In both Experiments 1A and 1B, we asked participants whether they had based their judgments on specific rules or strategies. Most participants reported basing their responses merely on whether a sequence felt familiar or similar. These verbal reports suggest that they had very little explicit knowledge concerning sequence legality. Several of the participants reported that they made their judgments on the basis of a simple rule (e.g., “Certain animal combinations were not allowed,” or “If certain combinations of animals were the same as the learning phase, I said ‘yes’.” However, none of the participants was able to report anything specific that could actually help him or her make a test decision. On the basis of these verbal reports, there appears to be little evidence that the participants were explicitly aware of the distinction between legal and illegal sequences, despite their above chance performance.

The results of Experiments 1A and 1B demonstrate that participants acquired knowledge of both the visual and auditory regularities under standard unimodal conditions. Next, we presented sequences of stimuli in both visual and auditory modalities simultaneously under dual-modality conditions either using a single grammar (Experiment 2) or a different grammar for each modality (Experiment 3). The data obtained from Experiment 1 were used as baseline levels of performance to compare to levels of performance in the dual-modality conditions.

## Experiment 2: Dual-Modality, Cross-Modal ISL With One Grammar

The primary goal of Experiment 2 was to examine the effect of redundant cross-modal information on ISL. More specifically, this experiment was designed to examine whether ISL would be enhanced when participants were presented with corresponding auditory and visual sequences that contained the same grammatical regularities. If there is no difference in learning between this type of cross-modal format and the unimodal baseline conditions, then this would suggest that the sequential regularities in each perceptual domain was processed independently of the other. Instead, if behavioral facilitation is observed, this would suggest that the visual and auditory inputs were integrated together to form a unified cross-modal representation of the sequential regularities.

### Method

#### Subjects

Fifty-two Chinese graduate students were recruited (Age range = 23–30; Mean age = 25.96; Females = 22). We excluded 2 subjects from analysis who failed to follow instructions. The priori power analysis showed, despite 2 participants’ data was excluded in hypothesis tests, the sample size of 50 > 42 was adequate to reject null hypothesis with power = 0.9 at alpha = 0.05 when small effect size of $\eta_{p}^{2}$ was greater than 0.1.

#### Materials

**FIGURE 3:**
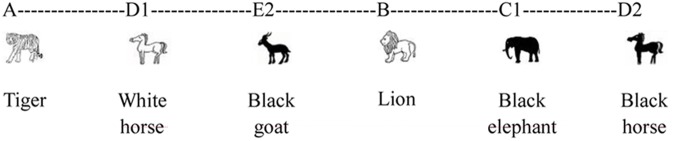
Sample of cross-modal stimuli presentation in Experiment 2. The animal pictures were the visual stimuli, and the animal names were the auditory stimuli presented in Chinese.

Visual stimuli was identical to Experiment 1A, and auditory stimuli was identical to Experiment 1B; visual and auditory sequences were produced by the same grammar as Experiment 1 and were presented simultaneously with each other. An example of cross-modal sequence is presented in **Figure 3**.

#### Procedure

In the learning phase, auditory and visual sequences were presented simultaneously, with the visual training set instantiated as animal pictures and audio training set as animal names. The auditory and visual sequences were identical and presented together during the familiarization phase (see **Figure 3**). For example, every time participants were presented with tiger in the visual modality, they also heard tiger in the auditory modality. At the beginning of the acquisition phase, participants were told nearly the identical story as provided in Experiments 1 and 2, with the additional mention that the animal pictures and names would be presented at the same time.

In the test phase, even though they were trained on a corresponding audiovisual sequence, participants were only tested on the pictures or on the sounds, as was the case in Experiment 1. Participants were randomly assigned to two groups, one group was tested on the visual stimuli, and the other group was tested on the auditory stimuli. The testing phase instructions were identical to the instructions used in Experiments 1 and 2.

### Results and Discussion

**FIGURE 4:**
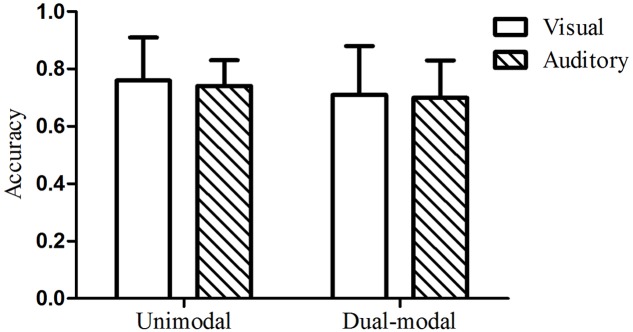
Mean test accuracy under redundant dual-modality conditions (Experiment 2) compared to unimodal controls (Experiment 1). The error bars represented the standard deviation of the mean to the figure.

The mean test accuracy of the visual group in Experiment 2 was 126 out of 160 (79%), with a standard deviation of 21. A one-sample *t-*test indicated that performance on the visual test was significantly above chance, *t*(24) = 11.27, *p* < 0.001, *d* = 2.23. The mean test accuracy of the auditory group was 115 out of 160 (72%), with a standard deviation of 25. A one-sample *t-*test indicated that performance on the auditory test was also significantly above chance, *t*(24) = 6.86, *p* < 0.001, *d* = 1.38. A two-way analysis of variance (ANOVA) with condition (dual-modal vs. unimodal baseline) and modality (visual vs. auditory) as between-participants factors was performed. There was no main effect of condition [*F*_(1,92)_ = 2.16, *p* = 0.15, $\eta_{p}^{2}$ = 0.023] or modality [*F*_(1,92)_ = 0.04, *p* = 0.85, $\eta_{p}^{2}$ < 0.001], nor was there a significant interaction [*F*_(1,92)_ = 0.34, *p* = 0.56, $\eta_{p}^{2}$ = 0.004]. These results indicated that there was no enhanced effect of ISL when audiovisual sequences with the same grammatical regularities were presented simultaneously (see **Figure 4**). The very small effect sizes indicate a substantial overlap in the score distributions across the two conditions. To reflect the extent to which the distributions separated across conditions with the effect of this size ($\eta_{p}^{2}$ = 0.023), we utilized a priori power analysis based on G^∗^Power version 3.1.9.2 package and found that 2088 participants in total were needed for an effect of this size to reach statistical significant We further calculated the Bayes Factor (BF) using the R package Bayes Factor (Morey and Rouder, 2015), supplementing the ANOVA, to compare the fit of the data under the null hypothesis and the alternative hypothesis (see von Koss Torkildsen et al., 2018 for a recent example of taking such an approach). The analyses indicated that the data are more in line with the null hypothesis (no difference between conditions), with a BF of 1.80 for the comparison of the null hypothesis over the alternative hypothesis (a difference between conditions) in contrast to a BF of 0.56 for the comparison of the alternative hypothesis over the null hypothesis, suggesting that the implicit learning performance under dual-modal condition are not differ from that under unimodal condition of either visual or auditory.

The test performance under redundant dual-modality conditions was identical to that under unimodal conditions, which suggests that the underlying learning systems operated in parallel and independently of one another. However, the strongest test of whether dual-modality ISL occurs independently is to present audio–visual sequences simultaneously but with each perceptual modality containing different grammatical regularities.

## Experiment 3: Dual-Modality, Cross-Modal ISL With Different Grammars

The primary goal of Experiment 3 was to examine whether ISL would be decreased when participants were presented with auditory and visual sequences simultaneously with each modality containing different grammatical regularities. If interference is observed, this would suggest that a single ISL system was operating over the cross-modal input, attempting to integrate the sequences into a coherent representation. On the other hand, if no decrement in performance is observed, this would provide strong evidence that the two sets of sequential regularities were processed and learned independently of one another.

### Method

#### Subjects

Forty-nine Chinese graduate students were recruited for the tests under dual-modality condition (Age range = 23–30; Mean age = 25.40; Females = 24). We excluded 1 participant from analysis who failed to follow instructions. An additional 24 Chinese graduate students were recruited to serve as unimodal baseline controls using the new auditory grammar that was used in Experiment 3 (Age range = 24–29; Mean age = 25.88; Females = 13). The power analysis, same as it used in Experiment 2, showed the sample size of 49 > 42 was well-advised to detect a small effect size of $\eta_{p}^{2}$ = 0.1 with power = 0.9 at alpha = 0.05.

#### Materials

**FIGURE 5:**
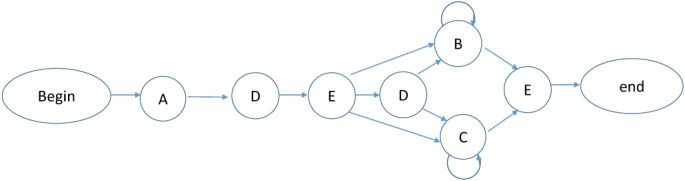
Artificial Grammar 2.

The materials were similar to the previous experiments, except that the auditory sequences were generated from a second grammar which was different from the grammar used for the visual sequences. The new grammar is presented in **Figure 5** and the new auditory sequences used in the acquisition and test phases are shown in Appendix A (Grammar 2) and Appendix C, respectively.

#### Procedure

**FIGURE 6:**
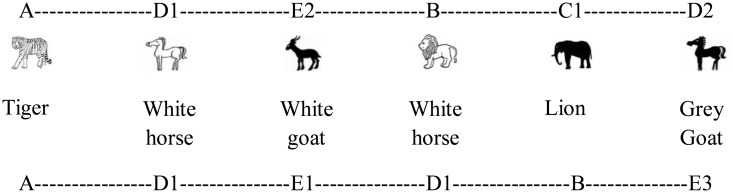
Sample of cross-modal stimuli presentation in Experiment 3. The animal pictures were the visual stimuli (generated from artificial grammar 1) and the animal names were the auditory stimuli (generated from artificial grammar 2) presented in Chinese.

The procedure was identical to Experiment 2, except for the different auditory sequences. For example, if participants were presented with tiger in the visual modality, it was very likely they heard the name of a different animal (such as lion) in the auditory modality because unlike Experiment 2, there was no cross-modal redundancy. **Figure 6** shows an example of cross-modal sequence used in Experiment 3. In the test phase, even though they were trained on cross-modal sequences, participants were only tested on the visual or auditory sequences, as was the case in Experiment 1. Participants were randomly assigned to two groups, one group was tested on the visual stimuli, and the other group was tested on the auditory stimuli. Instructions for the learning and test phases were identical to Experiment 2.

Because we used a new grammar for the auditory sequences in Experiment 3. It is necessary to obtain a new unimodal baseline. Thus we also collected the ISL performance of the new grammar under auditory unimodal conditions.

### Results and Discussion

**FIGURE 7:**
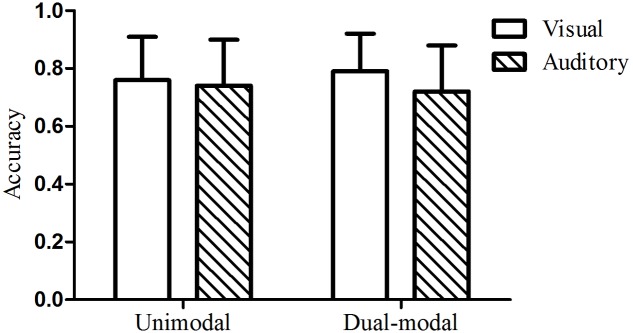
Mean test accuracy under dual-grammar, dual-modality conditions (Experiment 3) compared to their corresponding unimodal baseline condition. The error bars represented the standard deviation of the mean to the figure.

The mean test accuracy of the visual group in Experiment 3 was 114 out of 160 (71%), with a standard deviation of 27. A one-sample *t-*test indicated that performance on the visual test was significantly above chance, *t*(23) = 6.12, *p* < 0.001, *d* = 1.24. The mean test accuracy of the auditory group was 112 out of 160 (70%), with a standard deviation of 21. A one-sample *t-*test indicated that performance on the auditory test was also significantly above chance, *t*(23) = 7.70, *p* < 0.001, *d* = 1.54. The mean test accuracy of the new grammar under auditory unimodal conditions was 118 out of 160 (74%), with a standard deviation of 14. A two-way analysis of variance (ANOVA) with condition (dual-modal vs. unimodal baseline) and modality (visual vs. auditory) as between-participants factors was performed. There was no main effect of condition [*F*_(1,90)_ = 0.15, *p* = 0.70, $\eta_{p}^{2}$ = 0.002] or modality [*F*_(1,90)_ = 3.61, *p* = 0.06, $\eta_{p}^{2}$ = 0.039], nor was there a significant interaction [*F*_(1,90)_ = 0.02, *p* = 0.90, $\eta_{p}^{2}$ < 0.001]. The new auditory unimodal baseline from this experiment was used. These results indicated that there was no interference when audiovisual sequences with different grammatical regularities were presented simultaneously (see **Figure 7**). A power analysis showed that we would need 23694 participants in total for an effect of this size ($\eta_{p}^{2}$ = 0.002) to reach statistical significance. A Bayesian analysis also indicated that the data were more consistent with the null hypothesis of no difference between conditions, as indicated by a BF of 4.31, as opposed to a BF of 0.23 for the alternative hypothesis of a difference between conditions.

A one-way ANOVA (Experiment 1A visual baseline, Experiment 2 visual modality, and Experiment 3 visual modality) revealed no significant difference in visual test scores, *F*(2,68) = 1.97, *p* = 0.15, $\eta_{p}^{2}$ = 0.055. A power analysis showed that we would need 222 participants in total for an effect of this size to reach statistical significance. The BF analysis suggested that the data are more in line with the null hypothesis of no condition effect, as indicated by a BF of 1.74 for the comparison of the null hypothesis over the alternative hypothesis in contrast to a BF of 0.57 for the comparison of the alternative hypothesis over the null hypothesis.

A second one-way ANOVA (supplementing auditory baseline, Experiment 2 auditory modality, and Experiment 3 auditory modality) revealed no significant difference in auditory test scores across all experiments, *F*(2,70) = 0.85, *p* = 0.43, $\eta_{p}^{2}$ = 0.024. Learning under dual-stream conditions in both Experiment 2 (same grammar) and Experiment 3 (different grammars) had essentially the same levels of learning as under unimodal conditions, indicating that the cross-modal presentation format did not influence learning of each perceptual input stream. A power analysis showed that we would need 519 participants in each condition for an effect of this size to reach statistical significance. The BF analysis suggested that the data are more in line with the null hypothesis of no condition effect, as indicated by a BF of 2.84 for the comparison of the null hypothesis over the alternative hypothesis in contrast to a BF of 0.35 for the comparison of the alternative hypothesis over the null hypothesis. The fact that participants were able to simultaneously learn two different sets of sequential regularities from two different perceptual modalities is consistent with the involvement of independent and separate learning subsystems.

## General Discussion

The primary goal of this research was to test systematically whether dual-modality input streams would affect learning for each separate modality. In Experiment 1, we presented visual and auditory streams in isolation to provide baseline levels of unimodal performance used as comparisons for Experiments 2 and 3. In Experiment 2, we presented learners simultaneously with cross-modal streams that consisted of redundant information, that is, the identical sequences using the same artificial grammar. We found no effect on learning under such dual-stream conditions; participants were able to learn both visual and auditory streams successfully and the ISL effect was not enhanced relative to Experiment 1, the baseline level.

The nervous systems of many organisms have the ability to integrate perceptual inputs across different sensory modalities (e.g., visual and auditory), and then to determine the relationship between the corresponding inputs. This generally results in an enhancement for processing of the cross-modal information, allowing the organism to respond more accurately and quickly when processing multiple sources of sensory information from the same object at the same time (Liu et al., 2010). That there was no facilitative effect observed under redundant cross-modal conditions in Experiment 2 (using the pictures of animals paired with the auditory name of each) suggests that the sequential regularities were processed and learned independently within each perceptual modality, with no cross-modal integration occurring.

A complementary way to examine the effect of cross-modal input on ISL is to present learners with audiovisual streams containing different sequential regularities in each modality. This was explored in Experiment 3, in which we examined whether ISL would be decreased due to the interference between the two modalities. Incredibly, the results showed that this manipulation resulted in no decrement to performance relative to baseline learning conditions. That participants demonstrated learning of the sequential regularities for each input modality, despite each input stream providing different and conflicting information, is perhaps the strongest evidence yet that ISL consists of separate perceptual learning mechanisms operating in parallel (Conway and Christiansen, 2006; Frost et al., 2015).

If ISL is based on a common central mechanism, then dual-modality input conditions should result in cross-modal effects (either facilitation or interference, depending on the nature of the cross-modal relations). This was not observed. There was no difference in the levels of learning for either modality across any of the familiarization conditions (unimodal vs. cross-modal), suggesting that learning in each modality proceeded independently and did not impact each other. The existence of independent learning mechanisms is consistent with the findings of Jiménez and Vázquez (2011) who assessed whether sequence learning and contextual cueing learning could occur simultaneously. They found that it is possible to learn simultaneously about both context information and sequence information (consisting of a relatively complex sequence of targets and responses), thus suggesting that these two learning processes do not compete for a limited pool of central cognitive resources. Their findings provided support for the existence of multiple parallel learning subsystem. A complementary finding was recently observed by Walk and Conway (2016). Their study demonstrated that when visual and auditory stimuli were interleaved in a sequential presentation format, adults readily learned dependencies between stimuli within the same modality (visual–visual or auditory–auditory) but were unable to learn cross-modal sequential dependencies (auditory–visual or visual–auditory).

In contrast, a growing body of evidence has demonstrated cross-modal effects during ISL. For instance, Cunillera et al. (2010) showed that simultaneous visual information could improve auditory ISL if the visual cues were presented near transition boundaries (see also Sell and Kaschak, 2009; Mitchel and Weiss, 2010; Thiessen, 2010). Similarly, Mitchel et al. (2014) used the McGurk illusion to demonstrate that learners can integrate auditory and visual input during a statistical learning task. These studies seem to provide evidence counter to the lack of behavioral facilitation that we observed in Experiment 2. On the flip side, Mitchel and Weiss (2011) found that when triplet boundaries in visual and auditory input streams were desynchronized, learning was disrupted. This would appear to provide evidence counter to our Experiment 3, which revealed a lack of interference when the sequential regularities were decoupled.

It is possible that our experimental design was insufficient to detect any differences in learning across the different conditions. However, another reason for the differences in effects across studies is that ours used an artificial grammar, which involves learning and generalization of patterns to novel stimuli, unlike the paradigms used by the other studies reviewed above, which used the traditional triplet task, which does not involve generalization to new sequences but rather requires recognizing familiar triplets from unfamiliar ones. It is possible that learning in these simpler designs that do not involve generalization to new stimuli, reflects more explicit learning and memory processes (relying on the medial temporal lobe, Schapiro et al., 2014) whereas AGL also crucially involves striatal and frontal systems such as basal ganglia and prefrontal cortex (Conway and Pisoni, 2008), which are thought to comprise the procedural memory system (Ullman, 2004). Thus, future research needs to disentangle the role of task in obtaining some of these disparate cross-modal effects.

We should also point out that other AGL tasks, not dissimilar to the one used here, have demonstrated that learning can be disrupted under certain conditions. For instance, Hendricks et al. (2013) showed that when participants engaged in a working memory task concurrently with a standard AGL task, learning was disrupted if the working memory task occurred during the test phase or if the grammar learning task involved transfer to a new stimulus set. Similarly, in Experiment 3 of the Conway and Christiansen (2006) study, learning was disrupted when the stimuli in the two sets of grammars were perceptually similar. These findings show that in principle, the AGL task is sensitive to disruption under specific conditions; thus the lack of such interference effects observed here appears to be meaningful.

In summary, our study revealed, quite remarkably, that participants were just as adept at learning statistical regularities from two streams as from one. Importantly, participants were able to track simultaneously two sets of sequential regularities regardless of the cross-modal stimulus relationship (i.e., whether the two input streams were coupled or decoupled). These findings add to a growing set of findings that suggest that ISL consists of the involvement of multiple learning subsystems that can operate independently and in parallel. This work further suggests that under certain cross-modal input conditions, information is not integrated across perceptual modalities but instead remains distinct, allowing for a powerful set of resources to encode and represent the perceptual regularities that exist in the environment.

## Author Contributions

XL, XZ, and WS all contributed to the conception and design of this work and interpretation of data; the drafting and revising of the manuscript; and final approval of the version to be published. XL furthermore carried out the acquisition and analysis of the data. CC contributed to the interpretation of data; drafting and revising of the manuscript; and final approval of the version to be published. YL contributed to the interpretation of data; drafting and revising of the manuscript; and final approval of the version to be published during the response to the reviewers.

## Conflict of Interest Statement

The authors declare that the research was conducted in the absence of any commercial or financial relationships that could be construed as a potential conflict of interest.
