# Supplementary material for: Lack of Cross-Modal Effects in Dual-Modality Implicit Statistical Learning
Source: Front Psychol. 2018 Feb 27;9:146. doi: 10.3389/fpsyg.2018.00146 (PMC5835111; doi:10.3389/fpsyg.2018.00146)
Supplement: Supplementary file 1 [file Data_Sheet_1.docx]

Appendix A

| Training Items | | | | | | | | | | | | | | | | |
| --- | --- | --- | --- | --- | --- | --- | --- | --- | --- | --- | --- | --- | --- | --- | --- | --- |
| Grammar 1 | | | | | | |  |  |  | Grammar 2 | | | | | | |
| A | D1 | E1 | B | C2 | D3 |  |  |  |  | A | D1 | E1 | D1 | B | E1 |  |
| A | D1 | E2 | A | D2 |  |  |  |  |  | A | D1 | E1 | B | E1 |  |  |
| A | D1 | E2 | B | C1 | D1 |  |  |  |  | A | D1 | E1 | D1 | B | E2 |  |
| A | D1 | E2 | B | C1 | D2 |  |  |  |  | A | D1 | E1 | D1 | B | E3 |  |
| A | D1 | E2 | B | C2 | D1 |  |  |  |  | A | D1 | E1 | D2 | B | E1 |  |
| A | D1 | E2 | B | C2 | D3 |  |  |  |  | A | D1 | E1 | D2 | B | E2 |  |
| A | D1 | E3 | A | D3 |  |  |  |  |  | A | D2 | E2 | B | E2 |  |  |
| A | D1 | E3 | B | C1 | D1 |  |  |  |  | A | D1 | E2 | D2 | C1 | E1 |  |
| A | D1 | E3 | B | C1 | D2 |  |  |  |  | A | D1 | E2 | D2 | C1 | E2 |  |
| A | D1 | E3 | B | C2 | D3 |  |  |  |  | A | D1 | E2 | D2 | C1 | E3 |  |
| A | D2 | E1 | A | D1 |  |  |  |  |  | A | D1 | E1 | B | E2 |  |  |
| A | D2 | E1 | A | D3 |  |  |  |  |  | A | D1 | E1 | B | E3 |  |  |
| A | D2 | E1 | B | C1 | D2 |  |  |  |  | A | D3 | E2 | D3 | C1 | E3 |  |
| A | D2 | E1 | B | C1 | D3 |  |  |  |  | A | D3 | E3 | D1 | C1 | E1 |  |
| A | D2 | E1 | B | C2 | D1 |  |  |  |  | A | D3 | E3 | D1 | C1 | E2 |  |
| A | D2 | E1 | B | C2 | D3 |  |  |  |  | A | D3 | E3 | D1 | C1 | E3 |  |
| A | D2 | E2 | B | C2 | D3 |  |  |  |  | A | D3 | E3 | D2 | C1 | E1 |  |
| A | D2 | E3 | A | D1 |  |  |  |  |  | A | D2 | E1 | B | E2 |  |  |
| A | D2 | E3 | A | D2 |  |  |  |  |  | A | D2 | E1 | B | E3 |  |  |
| A | D2 | E3 | B | C1 | D3 |  |  |  |  | A | D3 | E3 | D2 | C1 | E2 |  |
| A | D2 | E3 | B | C2 | D2 |  |  |  |  | A | D3 | E3 | D2 | C1 | E3 |  |
| A | D2 | E3 | B | C2 | D3 |  |  |  |  | A | D3 | E3 | D3 | C1 | E1 |  |
| A | D3 | E1 | A | D3 |  |  |  |  |  | A | D3 | E3 | B | E2 |  |  |
| A | D3 | E1 | B | C1 | D2 |  |  |  |  | A | D2 | E1 | D2 | C1 | E3 |  |
| A | D3 | E1 | B | C2 | D1 |  |  |  |  | A | D2 | E1 | D3 | C1 | E1 |  |
| A | D3 | E1 | B | C2 | D3 |  |  |  |  | A | D2 | E1 | D3 | C1 | E2 |  |
| A | D3 | E2 | A | D2 |  |  |  |  |  | A | D3 | E2 | B | E3 |  |  |
| A | D3 | E2 | A | D3 |  |  |  |  |  | A | D3 | E3 | B | E1 |  |  |
| A | D3 | E2 | B | C1 | D1 |  |  |  |  | A | D2 | E1 | D1 | C2 | E1 |  |
| A | D3 | E2 | B | C2 | D1 |  |  |  |  | A | D2 | E1 | D1 | C2 | E2 |  |
| A | D3 | E2 | B | C2 | D2 |  |  |  |  | A | D2 | E1 | D1 | C2 | E3 |  |
| A | D3 | E3 | B | C2 | D3 |  |  |  |  | A | D2 | E1 | D2 | C2 | E1 |  |
| B | C1 | D1 | E1 | A | D1 |  |  |  |  | A | D2 | E1 | D2 | C2 | E2 |  |
| B | C1 | D1 | E1 | B | C1 | D1 |  |  |  | A | D2 | E3 | D3 | C2 | C2 | E2 |
| B | C1 | D1 | E1 | B | C1 | D3 |  |  |  | A | D2 | E3 | D3 | C2 | C2 | E3 |
| B | C1 | D1 | E1 | B | C2 | D1 |  |  |  | A | D3 | E1 | D1 | C2 | C2 | E1 |
| B | C1 | D1 | E1 | B | C2 | D3 |  |  |  | A | D3 | E1 | D1 | C2 | C2 | E2 |
| B | C2 | D1 | E1 | A | D1 |  |  |  |  | A | D1 | E3 | D3 | C2 | E3 |  |
| B | C2 | D1 | E1 | A | D2 |  |  |  |  | A | D1 | E3 | D3 | C2 | E2 |  |
| B | C2 | D1 | E1 | B | C1 | D1 |  |  |  | A | D3 | E3 | D3 | C2 | C2 | E1 |
| B | C2 | D1 | E1 | B | C1 | D2 |  |  |  | A | D3 | E3 | D3 | C2 | C2 | E2 |
| B | C2 | D1 | E1 | B | C2 | D2 |  |  |  | A | D3 | E3 | D3 | C2 | C2 | E3 |
| B | C2 | D1 | E2 | A | D1 |  |  |  |  | A | D1 | E3 | D2 | C2 | E2 |  |
| B | C2 | D1 | E2 | A | D3 |  |  |  |  | A | D1 | E3 | D2 | C2 | E3 |  |
| B | C2 | D1 | E3 | A | D1 |  |  |  |  | A | D1 | E3 | D3 | C2 | E1 |  |
| B | C2 | D1 | E3 | A | D3 |  |  |  |  | A | D2 | E3 | D1 | C2 | E1 |  |
| B | C2 | D2 | E3 | A | D3 |  |  |  |  | A | D2 | E3 | D1 | C2 | E2 |  |
| B | C2 | D2 | E3 | A | D2 |  |  |  |  | A | D2 | E3 | D1 | C2 | E3 |  |
|  |  |  |  |  |  |  |  |  |  |  |  |  |  |  |  |  |

*Note*. Grammar1 referred to the training items used in Experiment 1and Experiment 2, and training items (visual stimuli) used in Experiment 3. Grammar 2 referred to the training items (auditory stimuli) used in Experiment 3.

Appendix B

| Test Items | | | | | | | | | | | | | | | | |
| --- | --- | --- | --- | --- | --- | --- | --- | --- | --- | --- | --- | --- | --- | --- | --- | --- |
| Legal | | | | | | |  |  |  | Illegal | | | | | | |
| A | D1 | E1 | B | C2 | D2 |  |  |  |  | A | D1 | B | E1 | C2 | D2 |  |
| A | D1 | E2 | A | D1 |  |  |  |  |  | A | D1 | A | E2 | D1 |  |  |
| A | D1 | E2 | B | C1 | D3 |  |  |  |  | A | D1 | B | E2 | C1 | D3 |  |
| A | D1 | E2 | B | C2 | D2 |  |  |  |  | A | D1 | B | E2 | C2 | D2 |  |
| A | D1 | E3 | A | D1 |  |  |  |  |  | A | D1 | A | E3 | D1 |  |  |
| A | D1 | E3 | B | C1 | D3 |  |  |  |  | A | D1 | B | E3 | C1 | D3 |  |
| A | D1 | E3 | B | C2 | D2 |  |  |  |  | A | D1 | B | E3 | C2 | D2 |  |
| A | D2 | E1 | A | D2 |  |  |  |  |  | A | D2 | A | E1 | D2 |  |  |
| A | D2 | E1 | B | C1 | D1 |  |  |  |  | A | D2 | B | E1 | C1 | D1 |  |
| A | D2 | E1 | B | C2 | D2 |  |  |  |  | A | D2 | B | E1 | C2 | D2 |  |
| A | D2 | E2 | B | C2 | D1 |  |  |  |  | A | D2 | B | E2 | C2 | D1 |  |
| A | D2 | E3 | A | D3 |  |  |  |  |  | A | D2 | A | E3 | D3 |  |  |
| A | D2 | E3 | B | C1 | D2 |  |  |  |  | A | D2 | B | E3 | C1 | D2 |  |
| A | D2 | E3 | B | C2 | D1 |  |  |  |  | A | D2 | B | E3 | C2 | D1 |  |
| A | D3 | E1 | A | D1 |  |  |  |  |  | A | D3 | A | E1 | D1 |  |  |
| A | D3 | E1 | B | C1 | D1 |  |  |  |  | A | D3 | B | E1 | C1 | D1 |  |
| A | D3 | E1 | B | C2 | D2 |  |  |  |  | A | D3 | B | E1 | C2 | D2 |  |
| A | D3 | E2 | A | D1 |  |  |  |  |  | A | D3 | A | E2 | D1 |  |  |
| A | D3 | E2 | B | C1 | D3 |  |  |  |  | A | D3 | B | E2 | C1 | D3 |  |
| A | D3 | E2 | B | C2 | D3 |  |  |  |  | A | D3 | B | E2 | C2 | D3 |  |
| A | D3 | E3 | B | C2 | D2 |  |  |  |  | A | D3 | B | E3 | C2 | D2 |  |
| B | C1 | D1 | E1 | A | D3 |  |  |  |  | B | C1 | E1 | D1 | A | D3 |  |
| B | C1 | D1 | E1 | B | C1 | D2 |  |  |  | B | C1 | E1 | D1 | B | C1 | D2 |
| B | C1 | D1 | E1 | B | C2 | D2 |  |  |  | B | C1 | E1 | D1 | B | C2 | D2 |
| B | C2 | D1 | E1 | A | D3 |  |  |  |  | B | C2 | E1 | D1 | A | D3 |  |
| B | C2 | D1 | E1 | B | C1 | D3 |  |  |  | B | C2 | E1 | D1 | B | C1 | D3 |
| B | C2 | D1 | E1 | B | C2 | D1 |  |  |  | B | C2 | E1 | D1 | B | C2 | D1 |
| B | C2 | D1 | E2 | A | D2 |  |  |  |  | B | C2 | E2 | D1 | A | D2 |  |
| B | C2 | D1 | E3 | A | D2 |  |  |  |  | B | C2 | E3 | D1 | A | D2 |  |
| B | C2 | D2 | E3 | A | D1 |  |  |  |  | B | C2 | E3 | D2 | A | D1 |  |
| B | C1 | D1 | E2 | A | D1 |  |  |  |  | B | C1 | E2 | D1 | A | D1 |  |
| B | C1 | D1 | E2 | A | D2 |  |  |  |  | B | C1 | E2 | D1 | A | D2 |  |
| B | C1 | D1 | E2 | A | D3 |  |  |  |  | B | C1 | E2 | D1 | A | D3 |  |
| B | C1 | D1 | E2 | B | C1 | D1 |  |  |  | B | C1 | E2 | D1 | B | C1 | D1 |
| B | C1 | D1 | E2 | B | C1 | D2 |  |  |  | B | C1 | E2 | D1 | B | C1 | D2 |
| B | C1 | D1 | E2 | B | C1 | D3 |  |  |  | B | C1 | E2 | D1 | B | C1 | D3 |
| B | C1 | D1 | E2 | B | C2 | D1 |  |  |  | B | C1 | E2 | D1 | B | C2 | D1 |
| B | C2 | D2 | E3 | A | D1 |  |  |  |  | B | C2 | E3 | D2 | A | D1 |  |
| B | C1 | D1 | E2 | A | D1 |  |  |  |  | B | C1 | E2 | D1 | A | D1 |  |
| B | C1 | D1 | E2 | A | D2 |  |  |  |  | B | C1 | E2 | D1 | A | D2 |  |
| B | C1 | D1 | E2 | A | D3 |  |  |  |  | B | C1 | E2 | D1 | A | D3 |  |
| B | C1 | D1 | E2 | B | C1 | D1 |  |  |  | B | C1 | E2 | D1 | B | C1 | D1 |
| B | C1 | D1 | E2 | B | C1 | D2 |  |  |  | B | C1 | E2 | D1 | B | C1 | D2 |
| B | C1 | D1 | E2 | B | C1 | D3 |  |  |  | B | C1 | E2 | D1 | B | C1 | D3 |
| B | C1 | D1 | E2 | B | C2 | D1 |  |  |  | B | C1 | E2 | D1 | B | C2 | D1 |
| B | C2 | D2 | E3 | A | D1 |  |  |  |  | B | C2 | E3 | D2 | A | D1 |  |
| B | C1 | D1 | E2 | A | D1 |  |  |  |  | B | C1 | E2 | D1 | A | D1 |  |
| B | C1 | D1 | E2 | A | D2 |  |  |  |  | B | C1 | E2 | D1 | A | D2 |  |
| B | C1 | D1 | E2 | A | D3 |  |  |  |  | B | C1 | E2 | D1 | A | D3 |  |
| B | C1 | D1 | E2 | B | C1 | D1 |  |  |  | B | C1 | E2 | D1 | B | C1 | D1 |
| B | C1 | D1 | E2 | B | C1 | D2 |  |  |  | B | C1 | E2 | D1 | B | C1 | D2 |
| B | C1 | D1 | E2 | B | C1 | D3 |  |  |  | B | C1 | E2 | D1 | B | C1 | D3 |
| B | C1 | D1 | E2 | B | C2 | D1 |  |  |  | B | C1 | E2 | D1 | B | C2 | D1 |
| B | C2 | D2 | E3 | A | D1 |  |  |  |  | B | C2 | E3 | D2 | A | D1 |  |
| B | C1 | D1 | E2 | A | D1 |  |  |  |  | B | C1 | E2 | D1 | A | D1 |  |
| B | C1 | D1 | E2 | A | D2 |  |  |  |  | B | C1 | E2 | D1 | A | D2 |  |
| B | C1 | D1 | E2 | A | D3 |  |  |  |  | B | C1 | E2 | D1 | A | D3 |  |
| B | C1 | D1 | E2 | B | C1 | D1 |  |  |  | B | C1 | E2 | D1 | B | C1 | D1 |
| B | C1 | D1 | E2 | B | C1 | D2 |  |  |  | B | C1 | E2 | D1 | B | C1 | D2 |
| B | C1 | D1 | E2 | B | C1 | D3 |  |  |  | B | C1 | E2 | D1 | B | C1 | D3 |
| B | C1 | D1 | E2 | B | C2 | D1 |  |  |  | B | C1 | E2 | D1 | B | C2 | D1 |
| B | C2 | D2 | E3 | A | D1 |  |  |  |  | B | C2 | E3 | D2 | A | D1 |  |
| B | C1 | D1 | E2 | A | D1 |  |  |  |  | B | C1 | E2 | D1 | A | D1 |  |
| B | C1 | D1 | E2 | A | D2 |  |  |  |  | B | C1 | E2 | D1 | A | D2 |  |
| B | C2 | D2 | E3 | A | D1 |  |  |  |  | B | C2 | E3 | D2 | A | D1 |  |
| B | C1 | D1 | E2 | A | D1 |  |  |  |  | B | C1 | E2 | D1 | A | D1 |  |
| B | C1 | D1 | E2 | A | D2 |  |  |  |  | B | C1 | E2 | D1 | A | D2 |  |
| B | C1 | D1 | E2 | A | D3 |  |  |  |  | B | C1 | E2 | D1 | A | D3 |  |
| B | C1 | D1 | E2 | B | C1 | D1 |  |  |  | B | C1 | E2 | D1 | B | C1 | D1 |
| B | C1 | D1 | E2 | B | C1 | D2 |  |  |  | B | C1 | E2 | D1 | B | C1 | D2 |
| B | C1 | D1 | E2 | B | C1 | D3 |  |  |  | B | C1 | E2 | D1 | B | C1 | D3 |
| B | C1 | D1 | E2 | B | C2 | D1 |  |  |  | B | C1 | E2 | D1 | B | C2 | D1 |
| B | C2 | D2 | E3 | A | D1 |  |  |  |  | B | C2 | E3 | D2 | A | D1 |  |
| B | C2 | D2 | E3 | A | D1 |  |  |  |  | B | C2 | E3 | D2 | A | D1 |  |
| B | C1 | D1 | E2 | A | D1 |  |  |  |  | B | C1 | E2 | D1 | A | D1 |  |
| B | C1 | D1 | E2 | A | D2 |  |  |  |  | B | C1 | E2 | D1 | A | D2 |  |
| B | C1 | D1 | E2 | A | D3 |  |  |  |  | B | C1 | E2 | D1 | A | D3 |  |
| B | C1 | D1 | E2 | B | C1 | D1 |  |  |  | B | C1 | E2 | D1 | B | C1 | D1 |
| B | C1 | D1 | E2 | B | C1 | D2 |  |  |  | B | C1 | E2 | D1 | B | C1 | D2 |
| B | C1 | D1 | E2 | B | C1 | D3 |  |  |  | B | C1 | E2 | D1 | B | C1 | D3 |
|  |  |  |  |  |  |  |  |  |  |  |  |  |  |  |  |  |

*Note*. Test items used in Experiment 1, Experiment 2, Experiment 3 (visual stimuli).

Appendix C

| Test Items | | | | | | | | | | | | | | | | |
| --- | --- | --- | --- | --- | --- | --- | --- | --- | --- | --- | --- | --- | --- | --- | --- | --- |
| Legal | | | | | |  |  |  |  | Illegal | | | | | | |
| A | D1 | E1 | D2 | B | E3 |  |  |  |  | A | D1 | D2 | E1 | B | E3 |  |
| A | D2 | E2 | B | E1 |  |  |  |  |  | A | D2 | B | E2 | E1 |  |  |
| A | D1 | E1 | D3 | B | E1 |  |  |  |  | A | D1 | D3 | E1 | B | E1 |  |
| A | D1 | E1 | D3 | B | E2 |  |  |  |  | A | D1 | D3 | E1 | B | E2 |  |
| A | D2 | E2 | B | E2 |  |  |  |  |  | A | D2 | B | E2 | E2 |  |  |
| A | D1 | E1 | D3 | B | E3 |  |  |  |  | A | D1 | D3 | E1 | B | E3 |  |
| A | D1 | E2 | D1 | B | E1 |  |  |  |  | A | D1 | D1 | E2 | B | E1 |  |
| A | D2 | E2 | B | E3 |  |  |  |  |  | A | D2 | B | E2 | E3 |  |  |
| A | D1 | E1 | D3 | B | E3 |  |  |  |  | A | D1 | D3 | E1 | B | E3 |  |
| A | D1 | E2 | D1 | B | E1 |  |  |  |  | A | D1 | D1 | E2 | B | E1 |  |
| A | D1 | E2 | D1 | B | E2 |  |  |  |  | A | D1 | D1 | E2 | B | E2 |  |
| A | D1 | E1 | C1 | E1 |  |  |  |  |  | A | D1 | C1 | E1 | E1 |  |  |
| A | D3 | E3 | D3 | B | E2 |  |  |  |  | A | D3 | D3 | E3 | B | E2 |  |
| A | D3 | E3 | D3 | B | E3 |  |  |  |  | A | D3 | D3 | E3 | B | E3 |  |
| A | D3 | E2 | B | E2 |  |  |  |  |  | A | D3 | B | E2 | E2 |  |  |
| A | D3 | E2 | D3 | C1 | E1 |  |  |  |  | A | D3 | D3 | E2 | C1 | E1 |  |
| A | D3 | E2 | D3 | C1 | E2 |  |  |  |  | A | D3 | D3 | E2 | C1 | E2 |  |
| A | D1 | E1 | C1 | E3 |  |  |  |  |  | A | D1 | C1 | E1 | E3 |  |  |
| A | D3 | E2 | D2 | C1 | E1 |  |  |  |  | A | D3 | D2 | E2 | C1 | E1 |  |
| A | D3 | E2 | D2 | C1 | E2 |  |  |  |  | A | D3 | D2 | E2 | C1 | E2 |  |
| A | D3 | E2 | D2 | C1 | E3 |  |  |  |  | A | D3 | D2 | E2 | C1 | E3 |  |
| A | D3 | E2 | D1 | C1 | E3 |  |  |  |  | A | D3 | D1 | E2 | C1 | E3 |  |
| A | D1 | E1 | D2 | C1 | C1 | E1 |  |  |  | A | D1 | D2 | E1 | C1 | C1 | E1 |
| A | D1 | E1 | D2 | C1 | C1 | E2 |  |  |  | A | D1 | D2 | E1 | C1 | C1 | E2 |
| A | D3 | E1 | D3 | C1 | E3 |  |  |  |  | A | D3 | D3 | E1 | C1 | E3 |  |
| A | D1 | E1 | D1 | C1 | C1 | E2 |  |  |  | A | D1 | D1 | E1 | C1 | C1 | E2 |
| A | D1 | E1 | D1 | C1 | C1 | E3 |  |  |  | A | D1 | D1 | E1 | C1 | C1 | E3 |
| A | D3 | E2 | D1 | C1 | E1 |  |  |  |  | A | D3 | D1 | E2 | C1 | E1 |  |
| A | D3 | E2 | D1 | C1 | E2 |  |  |  |  | A | D3 | D1 | E2 | C1 | E2 |  |
| A | D3 | E2 | D1 | C1 | E3 |  |  |  |  | A | D3 | D1 | E2 | C1 | E3 |  |
| A | D3 | E1 | D2 | C1 | E2 |  |  |  |  | A | D3 | D2 | E1 | C1 | E2 |  |
| A | D3 | E1 | D2 | C1 | E1 |  |  |  |  | A | D3 | D2 | E1 | C1 | E1 |  |
| A | D2 | E2 | D3 | C1 | E1 |  |  |  |  | A | D2 | D3 | E2 | C1 | E1 |  |
| A | D2 | E1 | D1 | C1 | C1 | E1 |  |  |  | A | D2 | D1 | E1 | C1 | C1 | E1 |
| A | D1 | E1 | D2 | C1 | C1 | E1 |  |  |  | A | D1 | D2 | E1 | C1 | C1 | E1 |
| A | D1 | E1 | D2 | C1 | C1 | E2 |  |  |  | A | D1 | D2 | E1 | C1 | C1 | E2 |
| A | D1 | E2 | D1 | C1 | C1 | E1 |  |  |  | A | D1 | D1 | E2 | C1 | C1 | E1 |
| A | D3 | E1 | D2 | C1 | E3 |  |  |  |  | A | D3 | D2 | E1 | C1 | E3 |  |
| A | D3 | E1 | D3 | C1 | E1 |  |  |  |  | A | D3 | D3 | E1 | C1 | E1 |  |
| A | D3 | E1 | D3 | C1 | E2 |  |  |  |  | A | D3 | D3 | E1 | C1 | E2 |  |
| A | D3 | E1 | D3 | C1 | E3 |  |  |  |  | A | D3 | D3 | E1 | C1 | E3 |  |
| A | D1 | E1 | D2 | C1 | C1 | E3 |  |  |  | A | D1 | D2 | E1 | C1 | C1 | E3 |
| A | D1 | E1 | D3 | C1 | C1 | E1 |  |  |  | A | D1 | D3 | E1 | C1 | C1 | E1 |
| A | D1 | E1 | D3 | C1 | C1 | E2 |  |  |  | A | D1 | D3 | E1 | C1 | C1 | E2 |
| A | D1 | E1 | D3 | C1 | C1 | E3 |  |  |  | A | D1 | D3 | E1 | C1 | C1 | E3 |
| A | D2 | E3 | D2 | C1 | E1 |  |  |  |  | A | D2 | D2 | E3 | C1 | E1 |  |
| A | D2 | E3 | D2 | C1 | E2 |  |  |  |  | A | D2 | D2 | E3 | C1 | E2 |  |
| A | D2 | E3 | D2 | C1 | E3 |  |  |  |  | A | D2 | D2 | E3 | C1 | E3 |  |
| A | D2 | E3 | D3 | C2 | E1 |  |  |  |  | A | D2 | D3 | E3 | C2 | E1 |  |
| A | D3 | E1 | D3 | C2 | C2 | E1 |  |  |  | A | D3 | D3 | E1 | C2 | C2 | E1 |
| A | D3 | E1 | D3 | C2 | C2 | E2 |  |  |  | A | D3 | D3 | E1 | C2 | C2 | E2 |
| A | D3 | E1 | D3 | C2 | C2 | E3 |  |  |  | A | D3 | D3 | E1 | C2 | C2 | E3 |
| A | D3 | E2 | D1 | C2 | C2 | E1 |  |  |  | A | D3 | D1 | E2 | C2 | C2 | E1 |
| A | D2 | E3 | D3 | C2 | E2 |  |  |  |  | A | D2 | D3 | E3 | C2 | E2 |  |
| A | D2 | E3 | D3 | C2 | E3 |  |  |  |  | A | D2 | D3 | E3 | C2 | E3 |  |
| A | D3 | E1 | D1 | C2 | E1 |  |  |  |  | A | D3 | D1 | E1 | C2 | E1 |  |
| A | D3 | E1 | D1 | C2 | E2 |  |  |  |  | A | D3 | D1 | E1 | C2 | E2 |  |
| A | D2 | E1 | D1 | C2 | C2 | E2 |  |  |  | A | D2 | D1 | E1 | C2 | C2 | E2 |
| A | D2 | E1 | D1 | C2 | C2 | E3 |  |  |  | A | D2 | D1 | E1 | C2 | C2 | E3 |
| A | D2 | E1 | D2 | C2 | C2 | E1 |  |  |  | A | D2 | D2 | E1 | C2 | C2 | E1 |
| A | D2 | E1 | D2 | C2 | C2 | E2 |  |  |  | A | D2 | D2 | E1 | C2 | C2 | E2 |
| A | D2 | E1 | D3 | C2 | E3 |  |  |  |  | A | D2 | D3 | E1 | C2 | E3 |  |
| A | D2 | E2 | D1 | C2 | E1 |  |  |  |  | A | D2 | D1 | E2 | C2 | E1 |  |
| A | D2 | E2 | D1 | C2 | E2 |  |  |  |  | A | D2 | D1 | E2 | C2 | E2 |  |
| A | D2 | E2 | D1 | C2 | E3 |  |  |  |  | A | D2 | D1 | E2 | C2 | E3 |  |
| A | D2 | E2 | D2 | C2 | E1 |  |  |  |  | A | D2 | D2 | E2 | C2 | E1 |  |
| A | D2 | E2 | D3 | C2 | E2 |  |  |  |  | A | D2 | D3 | E2 | C2 | E2 |  |
| A | D2 | E2 | D3 | C2 | E3 |  |  |  |  | A | D2 | D3 | E2 | C2 | E3 |  |
| A | D1 | E3 | D3 | C2 | C2 | E2 |  |  |  | A | D1 | D3 | E3 | C2 | C2 | E2 |
| A | D1 | E3 | D3 | C2 | C2 | E3 |  |  |  | A | D1 | D3 | E3 | C2 | C2 | E3 |
| A | D1 | E3 | D2 | C2 | C2 | E3 |  |  |  | A | D1 | D2 | E3 | C2 | C2 | E3 |
| A | D1 | E3 | D3 | C2 | C2 | E1 |  |  |  | A | D1 | D3 | E3 | C2 | C2 | E1 |
| A | D2 | E3 | D3 | C2 | E2 |  |  |  |  | A | D2 | D3 | E3 | C2 | E2 |  |
| A | D2 | E3 | D3 | C2 | E3 |  |  |  |  | A | D2 | D3 | E3 | C2 | E3 |  |
| A | D3 | E1 | D1 | C2 | E1 |  |  |  |  | A | D3 | D1 | E1 | C2 | E1 |  |
| A | D3 | E1 | D1 | C2 | E2 |  |  |  |  | A | D3 | D1 | E1 | C2 | E2 |  |
| A | D3 | E1 | D1 | C2 | E3 |  |  |  |  | A | D3 | D1 | E1 | C2 | E3 |  |
| A | D3 | E1 | D2 | C2 | E1 | E3 |  |  |  | A | D3 | D2 | E1 | C2 | E1 | E3 |
| A | D1 | E3 | D2 | C2 | C2 | E1 |  |  |  | A | D1 | D2 | E3 | C2 | C2 | E1 |
| A | D1 | E3 | D2 | C2 | C2 | E2 |  |  |  | A | D1 | D2 | E3 | C2 | C2 | E2 |
|  |  |  |  |  |  |  |  |  |  |  |  |  |  |  |  |  |

*Note*. Test items used in Experiment 3 (auditory stimuli).
